# Primary pharyngeal synovial sarcoma in a pediatric patient: A case report

**DOI:** 10.1097/MD.0000000000028411

**Published:** 2021-12-30

**Authors:** Yun Jung Bae, Hyojin Kim, Wonjae Cha, Byung Se Choi

**Affiliations:** a Department of Radiology, Seongnam, Seoul National University College of Medicine, Seoul National University Bundang Hospital, Republic of Korea; b Department of Pathology, Seongnam, Seoul National University College of Medicine, Seoul National University Bundang Hospital, Republic of Korea; c Department of Otorhinolaryngology-Head and Neck Surgery, Seongnam, Seoul National University College of Medicine, Seoul National University Bundang Hospital, Republic of Korea.

**Keywords:** magnetic resonance imaging, oropharynx, synovial sarcoma

## Abstract

**Rationale:**

Synovial sarcoma is a rare malignant tumor that typically originates from the soft tissue of the extremities. The occurrence of primary pharyngeal synovial sarcoma is even rarer, and few studies have reported its radiological features. Here, we report a case of pediatric primary pharyngeal synovial sarcoma and describe the conventional and advanced magnetic resonance imaging (MRI) findings with pathologic correlation.

**Patient concerns:**

An 11-year-old girl presented to the otolaryngologic clinic with dysphagia.

**Diagnosis:**

Laryngoscopy revealed a large mass in the oropharynx. MRI revealed a well-defined soft tissue mass with a maximal diameter of approximately 5 cm originating from the submucosal space of the oropharynx. The mass was primarily solid and showed homogeneous contrast-enhancement. The mass was hypointense on T1-weighted images and hyperintense on T2-weighted images. The mass showed a homogeneously low apparent diffusion coefficient value on diffusion-weighted imaging, which indicated high tumor cellularity. Dynamic contrast-enhanced MRI revealed a hypovascular tumor with low values of the volume transfer constant between the extracellular extravascular space and blood plasma and blood plasma volume per unit tissue volume. Amide proton transfer-weighted MRI revealed a relatively high amide proton transfer signal in the tumor, indicating a high protein/peptide component. The patient underwent partial surgical resection of the tumor, and the diagnosis of biphasic synovial sarcoma was confirmed on postoperative pathological examination.

**Intervention:**

The patient was started on chemotherapy with vincristine, ifosfamide, doxorubicin, and etoposide.

**Outcomes:**

The tumor did not respond to the 3 cycles of the chemotherapy. Thus, the patient underwent second surgery and subsequent radiation therapy. The patient is now under ifosfamide/carboplatin/etoposide chemotherapy.

**Lesson:**

Synovial sarcoma should be considered in the differential diagnosis of pediatric oropharyngeal submucosal tumors. Multimodal MRI may aid diagnosis, although the final diagnosis should be based on the postoperative pathological examination findings.

## 1 Introduction

Synovial sarcoma is a rare malignant tumor of soft tissue origin that has a prevalence of approximately 10%.^[1]^ The most common sites of affection are the extremities, followed by the head and neck region.[^1^,^2^] In the head and neck region, it most commonly occurs in the paravertebral connective tissue and manifests as a retropharyngeal or parapharyngeal mass.^[2]^ Primary pharyngeal synovial sarcomas (PPSS) are rare, and only a few cases, including 1 case series, have been reported. Moreover, the characteristic imaging features of PPSS have not yet been determined. In this study, we report a case of pediatric PPSS and describe the findings of both conventional and advanced magnetic resonance imaging (MRI).

## 2 Clinical findings

This case report was approved by the institutional review board of Seoul National University Bundang Hospital (No. B-2106-690-701). Informed consent was obtained from the patient and parents. An 11-year-old girl presented to the department of head and neck surgery at our institution with a 1-month history of dysphagia. Laryngoscopy revealed a bulge and a huge yellowish mass at the right lateral pharyngeal wall. The mass seemed to cross the midline. The patient experienced snoring when lying down, but there was no sign of dyspnea.

**Figure 1:**
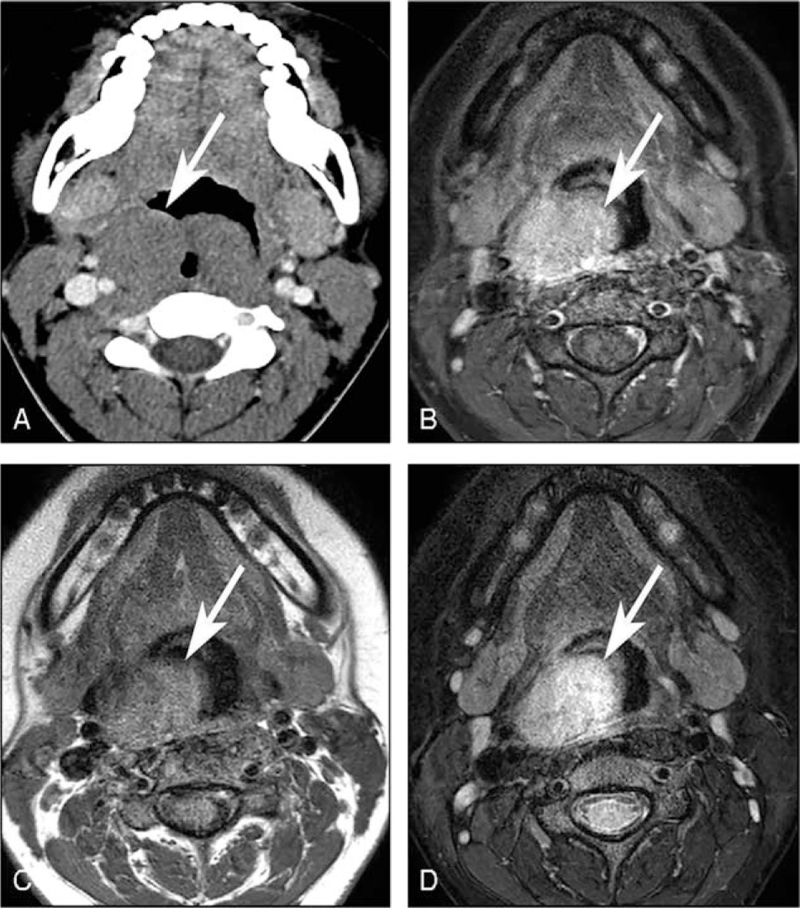
Computed tomography (CT) and conventional magnetic resonance imaging (MRI) findings. (A) CT and (B) postcontrast T1-weighted MRI images show a circumscribed solid mass with homogeneous contrast-enhancement in the oropharynx. (C) The mass shows low signal intensity on T1-weighted imaging. (D) The mass is hyperintense on fat-suppressed T2-weighted imaging. The white arrows indicate the mass.

**Figure 2:**
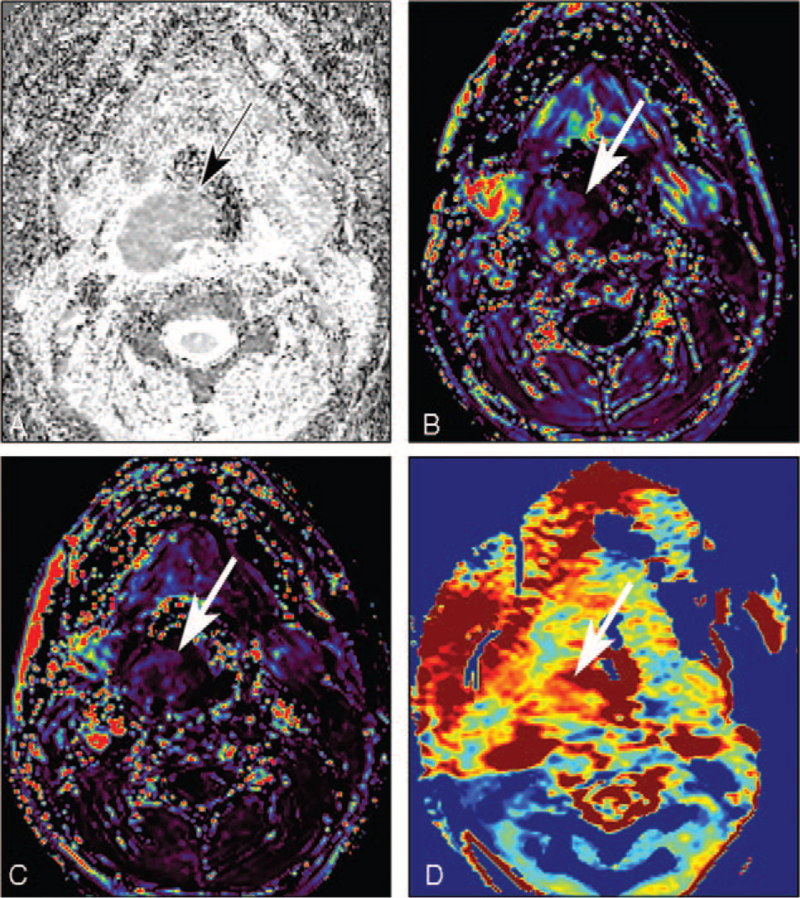
Advanced magnetic resonance imaging (MRI) findings. (A) The apparent diffusion coefficient (ADC) map derived from diffusion-weighted images shows a relatively low ADC value (0.76 × 10^−3^ mm^2^/s), indicating hypercellularity. (B, C) Maps derived from dynamic contrast-enhanced magnetic resonance images (MRI) show relatively low values of the volume transfer constant between the extracellular extravascular space and blood plasma (Ktrans, 0.04/min) and blood plasma volume per unit tissue volume (Vp, 0.004), which are characteristic of hypovascular tumors. (D) Amide proton transfer-weighted-MRI show a high amide proton transfer-weighted-signal of approximately 2.6%, suggesting a high protein/peptide content. The black arrow (A) and white arrows (B, C, D) indicate the mass.

Contrast-enhanced computed tomography (CT) and MRI were performed to identify the tumor. They revealed a 4.8 × 5.4 × 4.2 cm,^[3]^ circumscribed, solid mass with homogeneous contrast-enhancement in the oropharynx. The mass originated from the submucosal space, and the overlying mucosa was intact (Fig. 1A and B). On conventional MRI, the mass showed low signal intensity on T1-weighted images and high signal intensity on T2-weighted images (Fig. 1C and D). There was no evidence of cystic changes, necrosis, or hemorrhage in the mass. These imaging findings could not identify if this tumor was benign or malignant, and many differential diagnoses were considered, from benign submucosal schwannoma to malignant lymphoma or sarcoma. Thus, for further evaluation, the patient underwent advanced MRI, including diffusion-weighted imaging (DWI), dynamic contrast-enhanced magnetic resonance imaging (DCE-MRI), and amide proton transfer-weighted magnetic resonance imaging (APTw-MRI). On DWI, the mass showed a relatively low apparent diffusion coefficient (ADC) value of 0.76 × 10^−3^ mm^2^/s on the postprocessed ADC map, which was suggestive of high intra-tumoral cellularity (Fig. 2A). DCE-MRI revealed a contrast-enhancement pattern with initial enhancement and a late plateau. Evaluation of quantitative DCE-MRI parameters revealed relatively low values of the volume transfer constant between the extracellular extravascular space and blood plasma (Ktrans, 0.04/min) and blood plasma volume per unit tissue volume (Vp, 0.004), which are characteristics of hypovascular tumors (Fig. 2B and C). APTw-MRI revealed a high APTw signal of approximately 2.6%, which was suggestive of a high protein/peptide component (Fig. 2D).

**Figure 3:**
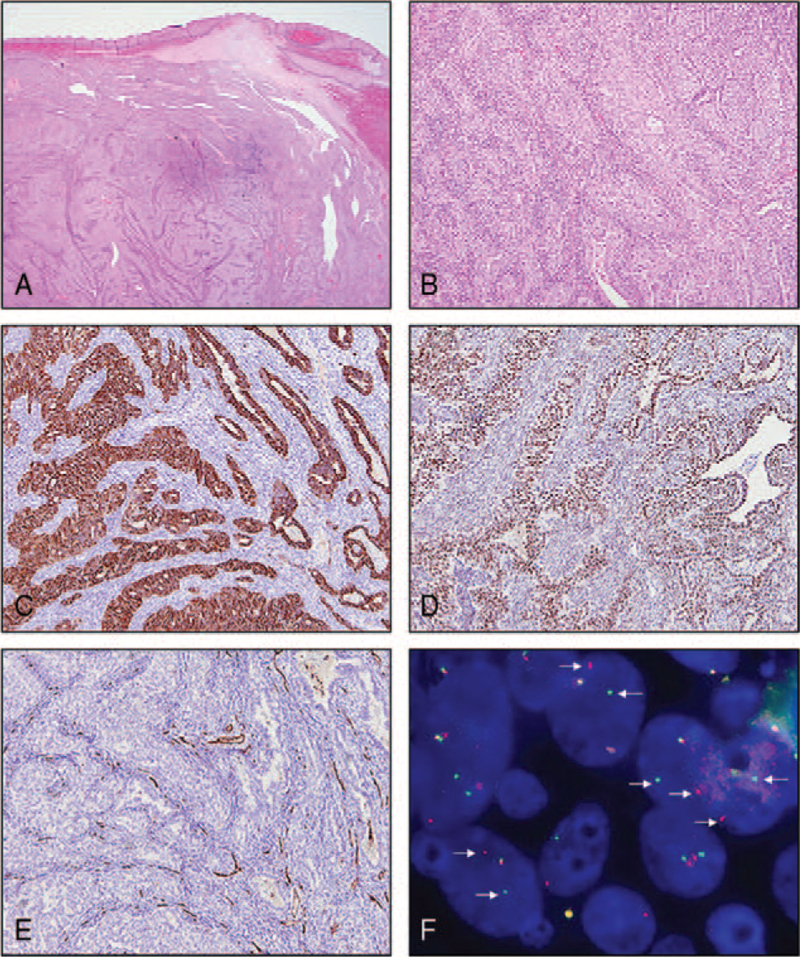
Microscopic and immunohistochemical findings. (A) The tumor is located in the subepithelial tissue and has a relatively well-demarcated margin. (B) It consists of epithelial and spindle cell components. (C) Cytokeratin and (D) transducin-like enhancer of split-1 show stronger expression in the epithelial component than in the spindle cell component. (E) CD34 expression and lower intra-tumoral microvascular density are seen. (F) Fluorescence in situ hybridization with a break-apart probe for *SYT* gene rearrangement reveals 1 fused signal and 1 separate red and green signal per nucleus, indicating the presence of a t(X;18) translocation. Magnification: 25×, 100×, 100×, 100×, 100×, and 1000× for (A), (B), (C), (D), (E), and (F), respectively.

To determine the pathologic diagnosis and relieve the airway obstruction, the patient underwent mass excision with a transoral approach. Intra-operatively, a yellowish firm mass was found attached to the right pharyngeal wall and partially resected into 2 pieces. Grossly, the 2 masses were smooth and firm and measured 4.1 × 2.4 × 1.3 cm^3^ and 3.9 × 3.3 × 2.4 cm^3^. Postoperative pathological examination revealed a circumscribed hypercellular lesion in the subepithelial tissue (Fig. 3A). The tumor consisted of epithelial and spindle cell components (Fig. 3B). The epithelial component was composed of columnar or cuboidal cells arranged in solid cords, nests, or gland-like formations, as seen in adenocarcinomas. The spindle cell component was present in the background and consisted of uniform fibroblast-like spindle cells with plump nuclei, as seen in fibrosarcomas. The epithelial and sarcomatous areas were intimately admixed. Immunohistochemical staining revealed that cytokeratin and transducin-like enhancer of split-1 were expressed more strongly in the epithelial component than in the spindle cell component (Fig. 3C and D). A low microvascular density (MVD) was observed on CD34 immunostaining (Fig. 3E). Fluorescence in situ hybridization revealed *SYT* gene rearrangement (Fig. 3F). The final diagnosis was biphasic synovial sarcoma.

On postoperative day 14, systemic chemotherapy was initiated for residual tumor. First cycle was constituted with vincristine (2 mg/d), ifosfamide (4.56 g/d), doxorubicin (30 mg/d), and etoposide (230 mg/d) for 3 days, and the patient received total 3 cycles of chemotherapy with 1-month of interval. During and after the chemotherapy, no adverse effect was reported. However, since the tumor did not show response to the chemotherapy, the patient underwent the second surgery for the tumor resection. After the surgery, subsequent radiation therapy with 52.5 Gy over 30 fractions for 2 months. Currently, the patient has started first cycle of the second-line systemic chemotherapy with ifosfamide (2175 mg), carboplatin (920 mg), and VP-16 (145 mg).

## 3 Discussion

Synovial sarcoma is a primitive mesenchymal malignant tumor with a poor prognosis that frequently occurs in adolescents and young adults.^[3]^ It generally arises near joints, but it can develop in unexpected locations such as the head and neck, heart, kidney, lung, and abdomen, which can result in a pre-operative misdiagnosis.[^4^,^5^,^6^,^7^] In the head and neck region, it most commonly occurs in the hypopharynx, but it can also occur in the prevertebral, parapharyngeal, laryngeal, and maxillofacial areas.[^8^,^9^] In addition, patients with synovial sarcoma involving the head and neck tend to be younger than those with synovial sarcoma of the extremities.[^8^,^9^] In such cases, various differential diagnoses should be considered, such as lymphoma, Ewing sarcoma, fibrosarcoma, hemangiopericytoma, and malignant peripheral nerve sheath tumor.[^10^,^11^]

Although pre-operative CT and MRI are necessary to ascertain tumor location, extent, and metastasis,[^11^,^12^] the imaging characteristics of PPSS have not been determined, presumably due to its rarity. In particular, MRI is superior to CT in assessing head and neck pathology because it provides a higher spatial resolution and contrast for soft tissue, and determining the MRI characteristics of PPSS will aid pre-operative diagnosis.

Here, we report the detailed conventional and advanced MRI characteristics of PPSS. Our patient's tumor showed low T1- and high T2-weighted signal intensities with homogeneous contrast-enhancement. These findings are consistent with those of a previous report.^[10]^ Notably, the T2-weighted signal intensity, which reflects intra-tumoral cellularity and water content, appeared hyperintense when compared with the adjacent muscles, thus ruling out lymphoma or Ewing sarcoma which have low to intermediate signal intensity.[^13^,^14^] Although PPSS, lymphoma, and Ewing sarcoma are all hypercellular tumors, biphasic synovial sarcoma has distinct epithelial and spindle cell components with prominent stromal matrices,^[2]^ while lymphoma and Ewing sarcoma have compact cellularity with less stromal prominence.[^13^,^14^] Consequently, lymphomas and Ewing sarcomas can have lower T2-weighted signal intensities than PPSS. Therefore, the T2-weighted signal intensity of the tumor can be used to differentiate PPSS from lymphoma or Ewing sarcoma.

We verified this assumption regarding tumor cellularity using quantitative DWI. DWI can be used to measure the differences in the random displacement of water molecules in tissues.^[15]^ This difference in water mobility is quantified by the ADC value, which is inversely correlated with tissue cellularity.^[15]^ ADC values have been known to be lower in malignant head and neck cancers than in benign tumors, with cut-off values of approximately 1.25 × 10^−3^ mm^2^/s. Furthermore, due to the compact cellularity of lymphomas, ADC values are lower in lymphomas than in squamous cell carcinomas, with a range of 0.64 to 0.66 × 10^−3^ mm^2^/s.^[16]^ Therefore, the ADC value of 0.76 × 10^−3^ mm^2^/s seen in our patient confirmed that the tumor had higher cellularity than a benign tumor, but lower cellularity than a lymphoma, which was a significant diagnostic clue.

Previous studies that have used DCE-MRI to assess head and neck tumors, adopted different vendors, scan protocols, and software, thus, the generalizability of the results could not be confirmed.^[17]^ However, we observed low values of Ktrans and Vp, both of which have been found to correlate with intra-tumoral MVD and vascular endothelial growth factor expression in pathologic studies.^[18]^ Interestingly, we observed low MVD on CD34 immunostaining and low intra-tumoral vascular parameters on DCE-MRI, indicating that DCE-MRI parameters can be used to demonstrate the hypovascularity of PPSS. This is consistent with the findings of a study of synovial sarcoma arising from the kidney.^[19]^ This might aid the differentiation of PPSS from other hypervascular tumors such as hemangiopericytoma. However, further research on the DCE-MRI parameters and their pathologic correlations is warranted.

Ours is the first study to report a high APTw-signal in a patient with PPSS. APTw-MRI is a recently developed molecular imaging technique that detects amide proton constituents in tumors based on chemical exchange saturation transfer between free water and mobile proteins/peptides.^[20]^ A few studies have used APTw-MRI to differentiate between benign and malignant head and neck tumors, and they found that a cutoff APTw-signal of approximately 2% showed a good performance in differentiating benign and malignant tumors.^[21]^ We observed a high APTw-signal (2.6%), which may be a characteristic finding of malignant tumors. However, no studies have compared APTw-signal values between lymphomatous and non-lymphomatous tumors. Further studies evaluating the importance of APTw-MRI for this differentiation are necessary.

In conclusion, PPSS is a rare tumor that is often challenging to diagnose and treat, and requires multidisciplinary management. Our patient demonstrated the characteristic findings of PPSS, including a homogeneously enhancing mass with a high signal intensity on T2-weighted conventional MRI, low ADC value on DWI, low Ktrans and Vp on DCE-MRI, and high APTw-signal on APTw-MRI. Considering these imaging findings in addition to the clinical features of PPSS could improve pre-operative diagnosis and enable appropriate surgical planning and early treatment.

## Acknowledgments

We would like to thank Editage (www.editage.co.kr) for English language editing.

## Author contributions

**Conceptualization:** Yun Jung Bae, Hyojin Kim, Wonjae Cha, Byung Se Choi.

**Data curation:** Yun Jung Bae, Hyojin Kim, Wonjae Cha, Byung Se Choi.

**Formal analysis:** Byung Se Choi.

**Investigation:** Yun Jung Bae, Hyojin Kim, Wonjae Cha, Byung Se Choi.

**Methodology:** Yun Jung Bae, Hyojin Kim, Wonjae Cha.

**Validation:** Wonjae Cha, Byung Se Choi.

**Visualization:** Yun Jung Bae, Hyojin Kim.

**Writing – original draft:** Yun Jung Bae, Hyojin Kim, Wonjae Cha, Byung Se Choi.

**Writing – review & editing:** Yun Jung Bae, Hyojin Kim, Wonjae Cha, Byung Se Choi.
